# Left Ventricular Unloading in Nonischemic Dilated Cardiomyopathy Improves Coronary Haemodynamic Reserve

**DOI:** 10.1002/ccd.31514

**Published:** 2025-03-27

**Authors:** Samer Fawaz, Rohini Ramaseshan, Sarosh Khan, John R. Davies, Carlos Collet, Grigoris V. Karamasis, Christopher M. Cook, Daniel A. Jones, Anthony Mathur, Thomas R. Keeble

**Affiliations:** ^1^ The Essex Cardiothoracic Centre Basildon Hospital Basildon UK; ^2^ Anglia Ruskin School of Medicine & MTRC Anglia Ruskin University Chelmsford UK; ^3^ Barts Heart Centre, Barts Health NHS Trust London UK; ^4^ Centre for Cardiovascular Medicine and Devices, William Harvey Research Institute Queen Mary University of London London UK; ^5^ Cardiovascular Center Aalst, OLV Clinic Aalst Belgium; ^6^ School of Medicine, Attikon University Hospital National and Kapodistrian University of Athens Athens Greece; ^7^ NIHR Barts Biomedical Research Centre Queen Mary University of London London UK

**Keywords:** cardiogenic, CS ‐ shock, ECMO/IABP/Tandem/Impella, HF ‐ heart failure, MCS ‐ mechanical circulatory support

## Abstract

**Background:**

The Impella CP is a catheter‐based ventricular assist device used in the management of cardiogenic shock and to support high‐risk percutaneous coronary interventions (PCI). Despite its growing use, the effects of Impella CP on coronary flow dynamics as measured by intracoronary continuous thermodilution have not been fully quantified.

**Aims:**

This study aimed to evaluate the impact of percutaneous ventricular unloading (PVU) with Impella CP on coronary flow reserve (CFR) and microvascular resistance reserve (MRR) in patients with severe nonischemic dilated cardiomyopathy undergoing intracoronary infusion of autologous bone‐marrow derived mononuclear cells (BMMNCs).

**Methods:**

Coronary flow (Q) was assessed using intracoronary continuous thermodilution both at rest (Qrest) and during hyperemia (Qhyper) before and after PVU with the Impella CP. Measurements were performed in the left anterior descending (LAD) artery using a dedicated pressure/thermistor coronary guidewire. CFR and MRR were calculated post‐hoc.

**Results:**

Nine patients underwent investigation with continuous thermodilution. Initiation of LV unloading with Impella CP resulted in a significant increase in CFR (from 2.72 ± 0.76, to 3.9 ± 1.84, *p* = 0.049) and MRR (from 3.09 ± 0.97, to 4.50 ± 1.93 *p* = 0.022). A decrease in mean Qrest and an increase in mean Qhyper was also noted.

**Conclusions:**

PVU with Impella CP led to a significant increase CFR and MRR, suggesting an improvement in coronary haemodynamic reserve. Further studies are needed to validate these findings in a larger patient population.

**Trial Registration:** Clinicaltrials. org: DCM Support NCT03572660.

## 1 Introduction

The Impella CP (Abiomed, USA) is a catheter‐based ventricular assist device commonly utilized in the management of cardiogenic shock and to support high‐risk percutaneous coronary interventions (PCI) [1]. It is typically delivered via large bore femoral artery access into the left ventricle where it aspirates and pumps blood into the ascending aorta at a rate between 2.5 and 5 L per minute. In addition to providing additional cardiac output, the Impella CP improves coronary haemodynamics by increasing distal coronary pressure [2], and by decreasing left ventricular end‐diastolic pressure and left ventricular wall stress [3].

## 2 Aims

This study sought to investigate coronary haemodynamics in nonischemic dilated cardiomyopathy (DCM) patients with severely impaired left ventricular function undergoing an intracoronary infusion of autologous bone‐marrow derived mononuclear cells (BMMNCs) with percutaneous left ventricular unloading (PVU) support with Impella CP. The effects on absolute coronary flow (Q) were measured both pre and post PVU using intracoronary continuous thermodilution, a method that allows the quantification of volumetric coronary flow (Q) both in resting (Q_rest_) and hyperemic conditions (Q_hyper_) [4], therefore permitting the calculation of coronary flow reserve (CFR) and microvascular resistance reserve (MRR), an index independent of epicardial stenosis severity [4]. Accordingly, this is the first study to quantify the effects of percutaneous LV unloading on CFR and MRR in DCM patients undergoing a high‐risk coronary intervention with severely impaired LV systolic function.

## 3 Methods

This study is a sub‐analysis of a trial (DCM Support—NCT03572660) investigating the effects of an intracoronary infusion of BMMNCs in patients with nonischemic DCM with severely impaired LV systolic function. All physiological measurements were performed in the Left Anterior Descending (LAD) artery.

The Impella CP device was used to facilitate infusion of the BMMNCs, as a prolonged balloon occlusion (3 min) of the coronary artery was needed for their delivery. A dedicated pressure/thermistor coronary guidewire (Pressure Wire X, Abbott, Boston, USA), in conjunction with a dedicated intracoronary infusion microcatheter (Rayflow ®, Hexacath, Paris, France) were used to acquire continuous thermodilution derived Q_rest_ and Q_hyper_, as well as distal coronary pressure (P_d_) and aortic pressure (P_a_). A single continuous thermodilution measurement was performed at baseline, followed by a further continuous thermodilution measurement after initiation of percutaneous LV unloading achieved with Impella at flow level P6. All measurements were performed before balloon occlusion and BMMNC infusion. CFR and MRR were calculated post‐hoc. Additional indices derived and calculated were microvascular resistance (R_µ_) at rest and hyperemia (R_µ, rest_ and R_µ, hyper_, respectively), and Fractional Flow Reserve (FFR).

## 4 Results

We studied nine patients who underwent continuous thermodilution assessment (mean age 58 ± 14, 55% female). All patients had dilated cardiomyopathies, of which there were varying aetiologies (4 idiopathic, 3 peripartum, 1 chemotherapy induced, and 1 genetic). The mean height and weight were 169 ± 7 cm and 104 ± 23 kg respectively. The mean ejection fraction was 25% ± 7%. All patients had non‐obstructive coronary artery disease, with a mean FFR of 0.90 ± 0.06. Due to the small sample size, P values should be interpreted with caution and the results considered hypothesis generating.

**Figure 1:**
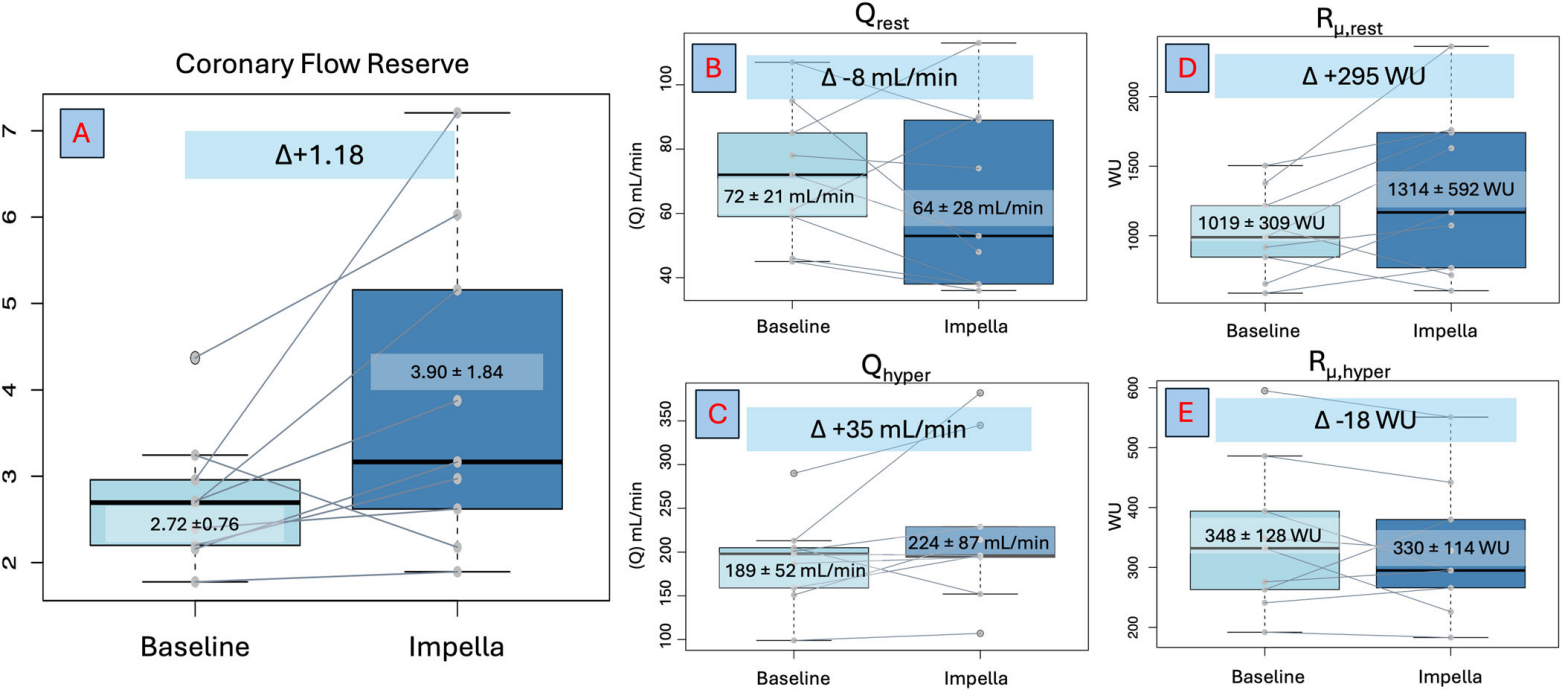
Box plots of continuous thermodilution indices with corresponding individual data points super‐imposed. A = Coronary flow Reserve. B = Q_rest_ – resting coronary flow. C = Q_hyper_ – hyperemic coronary flow. D = R_µ, rest_ – resting microvascular resistance. E = R_µ, hyper_ hyperemic microvascular resistance. [Color figure can be viewed at wileyonlinelibrary.com]

There was no difference in FFR at baseline and following PVU (0.90 ± 0.06 and 0.89 ± 0.03 respectively, *p* = 0.748). The initiation of LV support resulted in a significant increase in CFR (from 2.72 ± 0.76, to 3.9 ± 1.84, *p* = 0.049) (Figure 1), and MRR (from 3.09 ± 0.97, to 4.50 ± 1.93 *p* = 0.022). These findings are underpinned by changes in coronary flow and resistance parameters.

The initiation of LV unloading resulted a small reduction in mean Q_rest_ (72 ± 21 baseline and 64 ± 28 mL/min with support, mean difference (MD) −8 [95%CI −26–11] mL/min). This was associated with an increase resting P_a_ (P_a, rest_) (74 ± 8 to 77 ± 10 mmHg, MD 3 [95% CI −3–9] mmHg), and an increase in R_µ, rest_ (1019 ± 309 Woods Units (WU), to 1314 ± 592 WU, MD 295 [95% CI −30–621] WU) [5].

Conversely, LV unloading resulted in an increase in mean Q_hyper_ (189 ± 52 mL/min and baseline to 224 ± 87 mL/min with support, MD 35 [95%CI −12–82] mL/min). This was associated with an increase in hyperemic P_a_ (P_a, hyper_) (71 ± 9 to 78 ± 11 mmHg, MD 7 [95% CI 1–12] mmHg), with a clinically insignificant change in R_µ, hyper_ (348 ± 128 WU, to 330 ± 114 WU, MD −18 [95% CI −70–34] WU).

## 5 Discussion

The observed changes in coronary physiology following PVU align with prior mechanistic studies [2, 3, 5]. The reduction in Q_rest_ is minimal, with wide confidence intervals, which may reflect the highly variable nature of resting coronary flow. Accordingly, as Q_rest_ is subject to autoregulatory mechanisms, the increase in driving pressures (P_a_) are counteracted by an increase in R_µ, rest_ to maintain baseline coronary flow [5].

In contrast, Q_hyper_ changes more significantly following initiation of PVU. This increase in coronary flow is driven by an increase in driving pressures (P_a_) in the presence of maximally inhibited vascular resistance. No significant change in R_µ, hyper_ was noted, as the hyperemic state induced by the Rayflow catheter represents the physiological nadir in vascular resistance. Additionally, the observed rise in hyperemic flow may also be influenced by a reduction in LV end‐diastolic wall stress, as previously reported [3], although ventricular pressures were not measured in this study.

Overall, these findings suggest an increase in coronary haemodynamic reserve, as noted by the increase in CFR and MRR. Accordingly, this may provide a protective effect during high‐risk coronary interventions, where maintaining optimal myocardial perfusion is critical.

## 6 Limitations

There are several limitations in this study. First, this was a unique study population, with small sample size. Accordingly, the findings require validation in a larger cohort. Second, due to time constraints in delivering the BMMNCs before expiry, repeat measurements were not possible. However, the continuous thermodilution method has been shown to be highly reproducible in multiple key publications [6, 7]. Third, as the investigators were not blinded to which physiological traces were taken with or without LV unloading, it is not possible to rule out bias in interpreting the physiological traces.

## 7 Conclusions

In this limited case series of DCM patients, PVU led to a significant rise in CFR and MRR, suggesting an improvement in coronary haemodynamic reserve which may provide a protective effect during high‐risk coronary intervention. This effect is realized by a decrease in mean Q_rest_ and an increase in mean Q_hyper_. A larger study in a broader population group is needed to confirm these findings.

## Author Contributions

**Samer Fawaz:** conceptualization, data curation, formal analysis, investigation, methodology, visualization, writing – original draft preparation. **Rohini Ramaseshan:** conceptualization, data curation, formal analysis, investigation, methodology, visualization, writing – review and editing. **Sarosh Khan:** data curation, formal analysis, writing – review and editing. **John R. Davies:** supervision, visualization, writing – review and editing. **Carlos Collet:** formal analysis, methodology, writing – review and editing**. Grigoris V. Karamasis:** visualization, writing – review and editing. **Christopher M. Cook:** supervision, visualization, writing – review and editing. **Daniel A. Jones:** conceptualization, data curation, methodology, supervision, visualization, writing – review and editing. **Anthony Mathur:** conceptualization, data curation, formal analysis, funding acquisition, investigation, methodology, supervision, visualization, writing – review and editing. **Thomas R. Keeble:** conceptualization, methodology, supervision, writing – review and editing.

## Ethics Statement

All patients provided written informed consent, and the trial adhered to the principles of the Declaration of Helsinki.

## Conflicts of Interest

Dr Cook is a consultant for Philips Healthcare, Viz. ai. He receives speaker's fees from Boston Scientific and has equity in Cerebria. ai. Dr Keeble has received honoraria and institutional research funding from Abbott Vascular, Medtronic, Terumo, Zoll, and Shockwave. He has received consulting fees from BD and has received speaker fees from Cardionovum, Abbott Vascular, and Astra Zeneca. Dr Davies has received research grants from Abbott Vascular and Medtronic, and has received speaker fees from Boston Scientific. Dr Collett reports receiving research grants from Biosensor, Coroventis Research, Medis Medical Imaging, Pie Medical Imaging, CathWorks, Boston Scientific, Siemens, HeartFlow Inc, Abbott Vascular, and consultancy fees from HeartFlow Inc, OpSens, Abbott Vascular, and Philips Volcano. The other authors declare no conflicts of interest.

## Data Availability

The data that support the findings of this study are available from the corresponding author upon reasonable request.
